# Advanced case of PKDL due to delayed treatment: A rare case report

**DOI:** 10.1371/journal.pntd.0008052

**Published:** 2020-03-23

**Authors:** Roshan Kamal Topno, Vidya Nand Rabi Das, Maneesh Kumar, Major Madhukar, Krishna Pandey, Neena Verma, Kanhaiya Agrawal, Chandra Shekhar Lal, Niyamat Ali Siddiqui, Sanjiva Bimal, Pradeep Das

**Affiliations:** 1 Department of Epidemiology, ICMR-Rajendra Memorial Research Institute of Medical Sciences, Agamkuan, Patna, India; 2 Department of Clinical Medicine, ICMR-Rajendra Memorial Research Institute of Medical Sciences, Agamkuan, Patna, India; 3 Department of Virology, ICMR-Rajendra Memorial Research Institute of Medical Sciences, Agamkuan, Patna, India; 4 Department of Pathology, ICMR-Rajendra Memorial Research Institute of Medical Sciences, Agamkuan, Patna, India; 5 Department of Clinical Biochemistry, ICMR-Rajendra Memorial Research Institute of Medical Sciences, Agamkuan, Patna, India; 6 Department of Biostatistics, ICMR-Rajendra Memorial Research Institute of Medical Sciences, Agamkuan, Patna, India; 7 Department of Immunology, ICMR-Rajendra Memorial Research Institute of Medical Sciences, Agamkuan, Patna, India; 8 Department of Molecular Biology, ICMR-Rajendra Memorial Research Institute of Medical Sciences, Agamkuan, Patna, India; FIND, SWITZERLAND

## Abstract

Post-kala-azar dermal leishmaniasis (PKDL) is clinical outcome of visceral leishmaniasis (VL) and is thought to be the potential reservoir of parasite. Miltefosine (MF) is the only oral drug existing for treatment of post-kala-azar dermal leishmaniasis (PKDL). Increased miltefosine tolerance in clinical isolates of *Leishmania donovani* has been reported and is one of the major concerns in the treatment of PKDL. Here, we report a highly ulcerated PKDL case that was successfully cured after miltefosine treatment.

## Author summary

Post-kala-azar dermal leishmaniasis (PKDL) is a dermatological complication which usually occurs among treated VL cases. Despite active surveillance, some exclusive cases have been reported in the areas where VL is endemic. To eliminate VL, all source of reservoir to be managed properly. The appropriate diagnosis and treatment of PKDL cases are key. Here we report the identification and treatment of a highly ulcerated PKDL case. A detailed investigation inferred the possible role of CD8 + T cell in the skin ulcer. This report also describes how to identify the suspected case, to confirm the diagnosis, and to provide a successful treatment option.

## Ethics statement

The study was approved by the human ethics committee of Rajendra Memorial Research Institute of Medical Sciences, Agamkuan, Patna. Written informed consent was obtained before collecting the samples.

## Case

**Fig 1:**
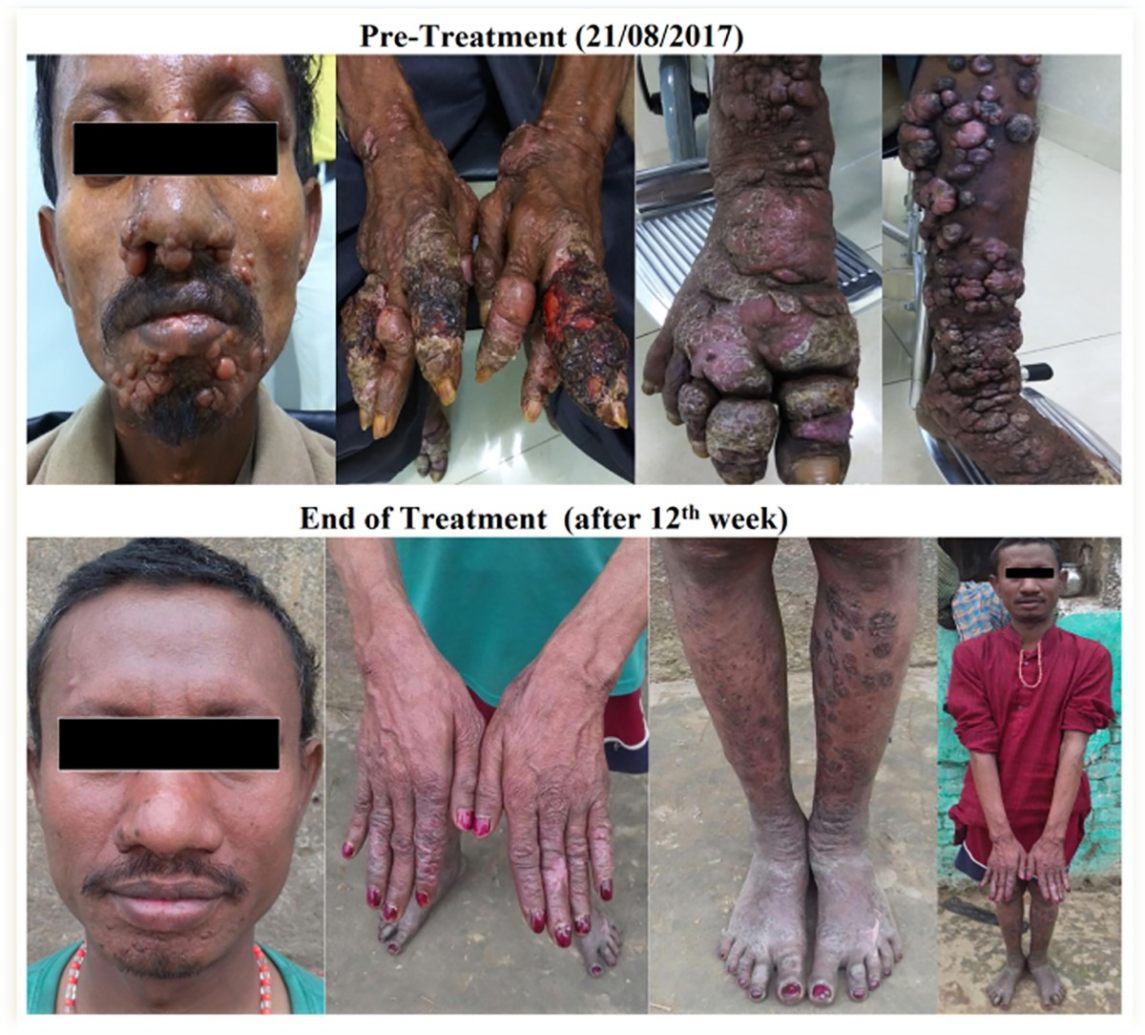
Different stages of PKDL appearance in the patient at pre-treatment and end of the treatment.

A Forty eight year-old male patient from Bahorchak, Begusarai district of Bihar, had been suffering from hypopigmented skin lesions on his face and limbs for 18 months. He visited nearby traditional healer with this dilemma, but due to lack of proper counselling and medication, the situation of the skin lesions remained the same. The patient had a VL history about 12 years ago and during that time he had received Sodium Antimony Gluconate (SAG) at a dose of 20mg/kg/body weight for one month. He was referred by a local clinician to ICMR- Rajendra Memorial Research Institute of Medical Sciences, Agamkuan at Patna, Bihar, India. He visited to our outdoor patient department (OPD) on 21^st^ August 2017. He was observed with nodular lesion on his chin and severely ulcerated lesions spread at the other parts of his body (Fig 1). On the physical examination, papulo-nodular skin lesions were found to be spread all over his body parts especially on his face, upper torso and on his upper and lower limbs. His lesions were found non-itching with intact sensitivity.

## Methods and materials

**Fig 2:**
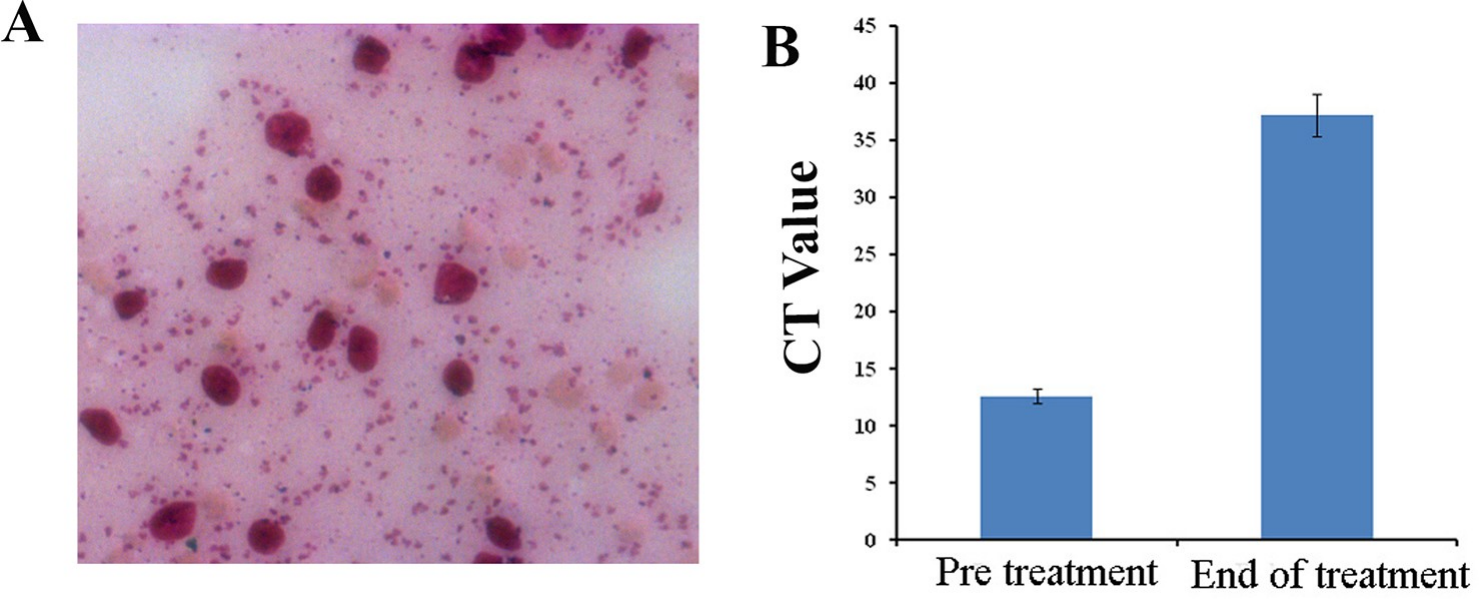
(A) Microscopic picture of LD bodies (5+) (B) Here Ct value is inversely proportional to Parasitic Load in pre treatment and end of the treatment. Parasitic load was found to be high in the pre-treatment condition as compared to the post treatment condition.

The patient then clinically diagnosed as PKDL positive with rK39 (InBios International, Inc. Seattle, USA) strip test followed by qPCR. Furthermore, the blood sample was collected for hematological and biochemical examinations, skin biopsy and Real Time PCR were performed to demonstrate the presence of Leishman-Donovan (LD) bodies and acid-fast bacilli (AFB). The Leishman-stained slit skin smear (SSS) was prepared from papulo-nodular skin lesions at the chin region of the patient, stained with Giemsa which was found to be positive (5+) for LD bodies (Fig 2A) [1, 2]. The skin biopsy was also performed with Ziehl Nielsen stains for AFB in order to detect leprosy which was found to be negative.

## Results

A support in this notion came from qPCR which was found to be positive for *Leishmania donovani* (**Fig 2(B)**). A lower Ct value was observed in qPCR at pre-treatment condition whereas a higher Ct value was observed in the post treatment condition. As the parasitic load is inversely proportional to the Ct value, the result depicts that treatment with miltefosine results parasite clearance from the highly ulcerated patient.

### Critical perspective of Post kala-azar dermal leishmaniasis (PKDL)

PKDL caused by *L*. *donovani* is an assorted dermatologic complication following apparent treatment of visceral leishmaniasis (VL). PKDL assumes significance as a major reservoir in inter-epidemic transmission of VL that could be possible through anthroponotic route. Besides an association with VL, a PKDL case has also been reported without any previous history of VL [3]. It clinically manifested with macular (hypo-pigmented patches), papulo-nodular lesions or polymorphic skin lesions.

### Pathological and Immunological investigation

Pathological investigations at intake revealed a total white blood cell count (WBC) of 4,700 cumm (ref. Value 4,000–11,000 cumm) with 64% neutrophils, 28% lymphocytes, 03% monocytes, and 05% eosinophils, haemoglobin 9.5 g/dL, platelet count 289,000/cumm, Aspartate aminotransferase test (ASAT) 56.8 units/L, alanine aminotransferase test (ALAT) 37.8, blood urea 17 mg/dL, serum creatinine 1.1 mg/dL, serum total bilirubin 0.99 mg/dL and random serum glucose 7.8 mmol/L. Immunologically, the patients was presented with 524/μl and 789/μl CD4 and CD8 as shown in a FACS-Caliber (BD, San Diego, USA) on cellQuest Pro-Software, respectively. The serological investigations were also performed for HIV (I & II), hepatitis-B and hepatitis C- virus which were found to be non-reactive.

### Treatment and progress

The treatment was started with Miltefosine at a dose of 50mg twice a day for 12 consecutive weeks. For additional wound management of the ulcerated lesions, ceftriaxone 1 gram was administered intravenously twice a day up to 10 days along with the miltefosine capsule. The patient was followed-up twice after 6^th^ week and 12^th^ week from his initial treatment, significant clinical enhancement have been noticed, without any side effect. Interestingly, a significant decline of the swollen nodular lesions of face and limbs was observed with the progress of treatment period. Follow-up slit skin smear taken at the end of treatment was found to be negative with LD bodies as well as RT-PCR. Interestingly, the CD4 and CD8 T cell count became 860/μl and 260/μl after treatment. After the end of treatment, other clinical observations were also found to be normal.

## Discussion

The chronic PKDL lesion is a well known dermatosis, often harbour parasite in the skin and plays a crucial role in the inter-epidemic communication for VL. The severe occurrence is immunologically intervened within the lesion [4, 5]. The skin lesions appear generally following treatment VL, after 2–3 years and in some cases, these lesions emerged even after a period of 10 years [6]. In this case report, the lesions exceeded the upper limit of time period for the appearance of PKDL. Our *in vitro* results demonstrated the presence of *Leishmania donovani* in skin slit smear. Though several PKDL cases appeared after the treatment of either AmBisome, miltefosine or other conventional drugs, but appearance of such ulcerated skin is rare. Therefore, we measured the level of CD4 as well as CD8 T cell at pre-treatment and at the end of the treatment. Previous studies also demonstrated the crucial role of CD8 T cell in immuno-pathogenesis with spectator of tissue damage [7, 8]. Furthermore, an exuberated CD8 T cells play vital role in immuno-pathogenesis and disease severity in PKDL as well as in other form of Leishmaniasis [7]. Indeed, patients suffered with PKDL, cutaneous and muco-cutaneous Leishmaniasis exhibited a significant increase in IL-10 producing CD8 T cell population which directly corroborate with disease pathogenesis [9, 10]. In corroboration with the previous studies, an elevated level of CD8 T cell at pre-treatment level is thought be involved in the highly ulcerated skin. Miltefosine is currently being used as anti-parasitic oral drug for treating PKDL which can easily accessible and safe to use [11, 12]. Previous study also demonstrated the treatment with miltefosine for 12 weeks (50 mg twice per day) has a cure rate of 93% (per protocol [PP] analysis) [13]. In Indian subcontinent, PKDL cases are treated according to national guidelines with miltefosine for 12 weeks with miltefosine. Interestingly, with the treatment of Miltefosine (50 mg twice per day for 84 days), the ulcerated skin became normal with reduced inflammation. Notably, during the treatment, no major alteration in biochemical as well as haematological parameters was observed (S1 Table). This report illustrates the success of treatment with miltefosine in treating highly ulcerated PKDL case without any severe complication. However, the adverse events described earlier are well-known and manageable with very minimum treatment facilities at the community level. Moving forward, this study will be useful to the medical practitioner as well as the policy makers to treat the severe ulcerated PKDL with Miltefosine.

## Supporting information

S1 Table (DOCX)

## References

[1] Chulay JD, Bryceson AD (1983) Quantitation of amastigotes of Leishmania donovani in smears of splenic aspirates from patients with visceral leishmaniasis. Am J Trop Med Hyg 32(3): 475–479. 10.4269/ajtmh.1983.32.475 6859397

[2] Das VN, Pandey K, Verma N, Lal CS, Bimal S, Topno RK, Singh D, Siddiqui NA, Verma RB, Das P (2009) Development of Post–Kala-Azar Dermal Leishmaniasis (PKDL) in Miltefosine-Treated Visceral Leishmaniasis. Am J Trop Med Hyg 80(3): 336–338. 19270277

[3] Verma N, Singh D, Pandey K, Das VN, Lal CS, Verma RB, Sinha PK, Das P (2013) Comparative evaluation of PCR and imprint smear microscopy analyses of skin biopsy specimens in diagnosis of macular, Papular, and mixed Papulo-nodular lesions of post-Kala-Azar dermal Leishmaniasis. J Clin Microbiol 51(12):4217–4219. 10.1128/JCM.01482-13 24068017 PMC3838087

[4] Mukhopadhyay D, Dalton JE, Kaye PM, Chatterjee M (2014) Post kala-azar dermal leishmaniasis: an unresolved mystery. Trends Parasitol 30(2):65–74. 10.1016/j.pt.2013.12.004 24388776 PMC3919212

[5] Salotra P, Singh R (2006) Challenges in the diagnosis of post kala-azar dermal leishmaniasis. Indian J Med Res 123(3): 295–310. 16778312

[6] Pandey K, Das VN, Singh D, Das S, Lal CS, Verma N, Bimal S, Topno RK, Siddiqui NA, Verma RB, Sinha PK (2012) Post-kala-azar dermal leishmaniasis in a patient treated with injectable paromomycin for visceral leishmaniasis in India. J Clin Microbiol 50(4):1478–1479. 10.1128/JCM.05966-11 22278840 PMC3318524

[7] Ganguly S, Das NK, Panja M, Pal S, Modak D, Rahaman M, Mallik S, Guha SK, Pramanik N, Goswami R, Barbhuiya JN (2008) Increased levels of interleukin-10 and IgG3 are hallmarks of Indian post-kala-azar dermal leishmaniasis. J Infect Dis 197(12):1762–1771. 10.1086/588387 18444882

[8] Zhang N, Bevan MJ (2011) CD8+ T cells: foot soldiers of the immune system. Immunity 35(2): 161–168. 10.1016/j.immuni.2011.07.010 21867926 PMC3303224

[9] Barral‐Netto M, Barral A, Brodskyn C, Carvalho EM, Reed SG (1995) Cytotoxicity in human mucosal and cutaneous leishmaniasis. Parasite Immunol 17(1): 21–28. 10.1111/j.1365-3024.1995.tb00962.x 7731732

[10] Brodskyn CI, Barral A, Boaventura V, Carvalho E, Barral-Netto M (1997) Parasite-driven in vitro human lymphocyte cytotoxicity against autologous infected macrophages from mucosal leishmaniasis. J Immun 159(9):4467–4473. 9379046

[11] Ramesh V, Singh R, Avishek K, Verma A, Deep DK, Verma S, Salotra P (2015) Decline in clinical efficacy of oral miltefosine in treatment of post kala-azar dermal leishmaniasis (PKDL) in India. PLoS Negl Trop Dis 22;9(10):e0004093. 10.1371/journal.pntd.0004093 PMC4619646 26492039

[12] Berger BA, Cossio A, Saravia NG, del Mar Castro M, Prada S, Bartlett AH, Pho MT (2017) Cost-effectiveness of meglumine antimoniate versus miltefosine caregiver DOT for the treatment of pediatric cutaneous leishmaniasis. PLoS Negl Trop Dis 11(4):e0005459 10.1371/journal.pntd.0005459 28384261 PMC5404883

[13] Zijlstra EE, Alves F, Rijal S, Arana B, Alvar J (2017) Post-kala-azar dermal leishmaniasis in the Indian subcontinent: A threat to the South-East Asia Region Kala-azar Elimination Programme. PLoS neglected tropical diseases. PLoS Negl Trop Dis 11(11):e0005877 10.1371/journal.pntd.0005877 29145397 PMC5689828

