# Supplementary material for: Advanced case of PKDL due to delayed treatment: A rare case report
Source: PLoS Negl Trop Dis. 2020 Mar 23;14(3):e0008052. doi: 10.1371/journal.pntd.0008052 (PMC7089400; doi:10.1371/journal.pntd.0008052)
Supplement: S1 Table — (DOCX) [file pntd.0008052.s001.docx]

|  | | | Before Treatment | Mid of the Treatment | End of The treatment |
| --- | --- | --- | --- | --- | --- |
| Total Leucocyte Count | | | 4,700 | 9,900 | 6,600 |
| Differential Leucocyte Count | **N** | | 64 | 55 | 41 |
|  | **L** | | 28 | 40 | 50 |
|  | **M** | | 03 | 02 | 03 |
|  | **E** | | 05 | 03 | 05 |
|  | **B** | | 00 | 00 | 01 |
| Hemoglobin gm % | | | 9.5 | 9.0 | 10.2 |
| Platelet Count | | | 283,000 | 276,000 | 272,000 |
| Total RBC | | | 4.29 | 4.23 | 4.29 |
| Serum Billirubin | | | 0.94 | 0.69 | 0.74 |
| SGOT | | | 56.8 | 34.0 | 30.3 |
| SGPT | | | 37.8 | 35.0 | 21.2 |
| Alkaline Phosphates | | | 115 | 119 | 84 |
| Blood Urea | | | 17 | 36 | 27 |
| Serum Creatinine | | | 1.1 | 1.0 | 0.8 |
| Serum Electrolyte | | **Na** | 140 | 141 | 140 |
|  |  | **K** | 4.5 | 4.7 | 4.7 |
| Skin Smear for LD Bodies | | | 5 + | Not Found | Not Found |
| HIV | | | Non Reactive | -- | -- |
| Hepatitis B | | | Non Reactive | **--** | **--** |
| Hepatitis C | | | Non Reactive | **--** | **--** |

**Table S1.** Detailed evaluation of biochemical as well as hematological parameters at pre-treatment, mid treatment and at the end of the treatment. No major alteration in the biochemical as well hematological as parameters was observed during the treatment with miltefosine.
